# Reimagining food security through income security: the potential role of universal basic income in the UK

**DOI:** 10.3389/fnut.2026.1816550

**Published:** 2026-07-27

**Authors:** Gemma Bridge, Remco Peters, Francis K. Poitier, Dave J. Beck

**Affiliations:** 1 Health and Wellbeing Research Centre, London South Bank University, London, United Kingdom; 2 School of Policy Studies, University of Bristol, Bristol, United Kingdom; 3 School of Medicine, University of Leeds, Leeds, United Kingdom; 4 School of Health and Society, University of Salford, Salford, United Kingdom

**Keywords:** cash transfers, dietary autonomy, financial precarity, food banks, food insecurity, food security, food vouchers, income security, universal basic income

## Abstract

Food insecurity is increasing across the United Kingdom, driven by rising living costs, welfare retrenchment, and increasingly insecure incomes. Current policy responses largely rely on targeted food assistance and emergency provision, which can mitigate acute hardship but operate downstream of the structural drivers of food insecurity. This article synthesises evidence from food-specific interventions, means-tested benefits, labour-market policies, and unconditional cash transfer programmes to examine whether Universal Basic Income (UBI) could address upstream determinants of food security in high-income contexts. Drawing on international and UK-relevant evidence, we argue that income security is a necessary precondition for effective food and nutrition policy. While UBI cannot resolve structural power imbalances in food systems or replace targeted nutrition interventions, it may reduce financial precarity, improve dietary autonomy, and enhance the effectiveness of existing policies. We conclude that UBI should be understood not as a standalone solution, but as a foundational intervention within integrated food, welfare, and public health strategies.

## 1 Introduction: the UK’S food insecurity crisis and policy turning point

Globally, tightening of welfare budgets have intensified debates about how best to support households facing rising living costs. Food insecurity, defined as the lack of reliable access to desirable, sufficient, safe, and nutritious food, has become an increasingly visible manifestation of this pressure (1). In the United Kingdom (UK), recent estimates suggest that 10 per cent of households are food insecure, with low-income and disabled groups at disproportionately high risk (2). Notably, over a third of households in receipt of Universal Credit report experiencing food insecurity (3).

The consequences of food insecurity go beyond hunger. Diets constrained by inadequate income are associated with higher risks of malnutrition, obesity, and non-communicable diseases, while the stress, stigma, and uncertainty of food insecurity undermine mental health, dignity, and social participation (4). Food insecurity is therefore not only a nutritional issue but a public health and social justice concern, shaped by upstream determinants such as income adequacy and welfare design.

Contemporary food policy responses to food insecurity have largely focused on targeted food assistance such as food banks and vouchers. While these interventions can mitigate acute hardship and, in some cases, improve nutritional outcomes, they operate downstream of the structural drivers of food insecurity—leaving individuals to continue to struggle with insecure employment or delayed benefit payments. Such drivers can lead to increased stress which in turn affects cognitive bandwidth, planning capacity, and emotional regulation—influencing food purchasing and preparation practices. Under such conditions, individuals can enter a *‘poverty mindset’* (5) whereby short-term coping strategies, such as buying cheaper, highly processed foods or reducing meal frequency, become rational responses to financial precarity. Such a mindset can in turn lead to poorer food intake, and poorer health outcomes—negatively impacting the very outcomes that targeted food assistance aims to achieve.

A predictable income, for example achieved through cash-first policy approaches, can disrupt this cycle. By reducing uncertainty and financial strain, a stable income floor may improve households’ ability to plan meals and invest in healthier food over time. Evidence on unconditional cash transfers is strongest in low- and middle-income countries (LMICs) where programmes have demonstrated improvements in food consumption and dietary diversity (6–8). Whilst the relevance of cash transfer to high-income welfare states remains underexplored, there is growing interest in their relevance. For example, the Department for Work and Pensions (DWP) recently endorsed a cash first approach to reduce food insecurity in England (9).

Universal Basic Income (UBI) (otherwise referred to as ‘Basic Income’, ‘Citizen’s Basic Income’, or ‘Citizen’s Income’) is a regular, unconditional cash payment to all individuals (10). With growing evidence that UBI could transform welfare and give communities agency, the approach is gaining growing attention (11). However, food and nutrition outcomes of UBI pilots have been under explored, hampering public health assessments. As the UK, and other high-income countries, confront persistent and widening food insecurity, understanding whether and how a UBI could address the upstream determinants of food security is timely and necessary.

This article explores the potential role of UBI in supporting food security by synthesising evidence from food-specific interventions, means-tested benefit studies, labour-market policies, and unconditional cash transfer programmes. In doing so, our article aims to inform current debates on welfare reform, food system transformation, and pathways towards nutrition security in the UK.

## 2 Limitations of current policy responses: necessary but insufficient ‘plasters’

Despite growing recognition of food insecurity as a public health concern, responses remain fragmented and reactive—focusing on mitigating acute need and operating downstream of the structural drivers of food insecurity. Such responses are limited in their capacity to deliver sustained improvements in dietary adequacy and food security because as long as household incomes remain unstable, inadequate, or conditional, food insecurity will persist.

### 2.1 Emergency food aid: food banks as stopgaps, not solutions

Emergency food aid, such as that offered by food banks, has become a central feature of the response to food insecurity, providing short-term relief during periods of crisis. Emergency food aid addresses the visible symptoms of food insecurity, for example, by offering individuals and households with food parcels to last up to a week, but does not change the conditions that precipitate food insecurity—insufficient and unstable incomes, housing costs and debt (12, 13). Nor does emergency food aid support wider nutritional goals, with food parcels often having limited nutritional quality and variety (14, 15).

Emergency food aid has also faced widespread criticism. Limited operating hours, hard to reach locations, and referral requirements make access challenging (16), whilst increasing experiences of stigma and shame amongst recipients (17). Adding to this, the sustainability of emergency food aid as a solution to food insecurity is in question, with food donations and surplus food diminishing (18). These limitations support the need for a ‘more than food aid’ approach, emphasising the need for holistic support, including sufficient income to access adequate food and housing [(19), p. 122].

### 2.2 Targeted food vouchers: constrained reach and uneven access

A longer-term approach to food insecurity could be delivered through targeted food voucher schemes, such as the UK’s Healthy Start programme. Such programmes aim to improve maternal and child nutrition by subsidising specific foods. Whilst research suggests that voucher programmes can support dietary quality among those who successfully access them (20–22), their effectiveness is constrained by low uptake, narrow and complex eligibility criteria (e.g., in terms of income levels), administrative barriers, and digital exclusion, particularly among already marginalised groups (23). Moreover, by design, these schemes restrict choice and operate within narrowly defined nutritional parameters, which may not align with household preferences or cultural food practices.

### 2.3 Subsidised food provision: child nutrition without household security

Subsidised food programmes, such as free school meals, represent one of the most robust and well-evidenced interventions for improving child nutrition and educational outcomes (24). By providing a guaranteed nutritious meal during the school day, these programmes reduce short-term hunger and support children’s health (24). However, their reach is inherently limited to specific age groups and settings, leaving household-level food insecurity largely unaddressed.

Families may continue to experience significant food hardship outside school hours, particularly during evenings, weekends, and school holidays. Moreover, eligibility thresholds and implementation challenges have drawn criticism for excluding families in precarious but not formally qualifying circumstances (25–27).

### 2.4 Means-tested benefits: instability within the safety net

Means-tested, behavioural conditional benefits, including Universal Credit (UC), are intended to provide a minimum standard of living for all members of a household. In practice, however, there are payment delays, sanctions, fluctuating entitlements, and administrative complexity that can exacerbate rather than alleviate food insecurity (28, 29). Conditionality and compliance requirements introduce income volatility, making it difficult for households to plan food budgets or maintain consistent dietary patterns. Fear of sanctions and stigma associated with claiming benefits further contribute to underclaiming among eligible individuals (30). Rather than functioning as a stable income floor, means-tested systems produce uncertainty and stress, reinforcing the very income insecurity that drives food hardship.

### 2.5 Minimum wage increases: partial protection through employment

Minimum wage increases are frequently proposed as a mechanism to improve living standards and reduce food insecurity among working households—whilst also maintaining choice and autonomy. However, while higher wage floors can increase purchasing power for those in stable employment, their benefits are unevenly distributed. Individuals unable to engage in paid labour due to disability, caring responsibilities, or insecure labour market attachment remain excluded, and in-work poverty persists even in contexts with statutory minimum wages. As such, labour-market interventions alone cannot guarantee nutrition security across the population.

### 2.6 Unconditional cash transfers: emerging evidence for income-led solutions

Studies from high income countries suggest that stabilising income, rather than regulating food choices, can improve food security by addressing its root causes. For example, participants in Ontario’s cancelled basic income pilot in Canada reported improved diet quality and reduced reliance on food banks (31), while US guaranteed income pilots have documented rapid reductions in food insecurity (32).

In low- and middle-income settings, UBI-style trials, where every resident of a specific area or community receives a basic income, have shown that individuals and households receiving unconditional income increase spending on food, diversify diets, and invest in productive assets without evidence of harmful inflationary effects. Examples include long-term trials in Uganda, Kenya, Namibia’s Basic Income Grant pilot (33), and India’s Madhya Pradesh experiment (34).

## 3 Could UBI serve as an upstream intervention?

### 3.1 What could a UBI achieve?

Food insecurity is a cross-cutting policy issue that spans multiple domains, including welfare, health, housing, employment, and education (28). In the UK, no single government department holds statutory responsibility for food insecurity, resulting in a fragmented policy landscape with overlapping but poorly coordinated interventions (29). This institutional fragmentation makes narrowly targeted solutions difficult to scale and sustain.

UBI is particularly compelling in this context because it addresses food insecurity by targeting inadequate and unstable income. As UBI does not fluctuate with employment status, household composition, or behavioural compliance, it offers predictability and stability, which in turn can have positive impacts on households’ capacity to budget and increase the ability to purchase adequate and nutritious food (35). Also compelling is the potential for actually simplifying administrative processes and costs—unlike UC, which was initially implemented to do just this, but as it has been delivered in different ways according to personal status and geography, simplicity and cost saving has not been realised (36).

UBI is underpinned by principles of universality, autonomy, and unconditionality. By removing eligibility assessments and conditionality, UBI simplifies welfare delivery, and reduces the psychological burden associated with means-tested systems. In doing so, UBI may reduce stress that can undermine both wellbeing and dietary practices and potentially improve reach and uptake of support among groups currently excluded by administrative complexity.

UBI also aligns closely with principles of food justice by supporting autonomy and dignity (37). Unlike vouchers or subsidies that constrain spending choices, UBI enhances autonomy and dignity by allowing individuals to decide how best to meet their own needs. This shift from paternalistic to trust-based support may strengthen social inclusion and reduce the stigma often associated with welfare receipt.

### 3.2 What are the limitations of a UBI in supporting improved food security?

Despite the opportunities of UBI, it is equally important to examine what UBI cannot achieve on its own.

UBI cannot directly alter food environments. For example, it would not increase the availability or affordability of healthy foods. This is important as rising food costs directly constrain purchasing power and have been shown to mediate the relationship between income, food insecurity, and health expenditure (38).

UBI is also unlikely to impact structural power imbalances within agri-food supply chains, such as corporate concentration or low farm incomes (39). While increased purchasing power, achieved by increasing available income within communities, may influence demand over time, these supply-side issues require complementary regulatory and food system interventions.

Similarly, UBI cannot resolve other major cost pressures that compete with food budgets, such as housing affordability, childcare costs, or unmanageable debt (35).

UBI should not be seen as a substitute for nutrition-specific interventions aimed at groups with distinct physiological needs, including children, pregnant women, and those with clinical nutritional requirements. Rather, it operates as a foundational layer upon which targeted supports can function more effectively.

## 4 UBI as a complement to other policies—a systems perspective

Debates around UBI are often framed in oppositional terms, positioning UBI as either a substitute for or a competitor to existing welfare, nutrition, or labour-market interventions. However, rather than replacing targeted policies, UBI may be more productively understood as a foundational intervention that enhances their effectiveness by addressing a core upstream determinant: income insecurity. By stabilising household resources, UBI can improve the conditions under which individuals are able to engage with, benefit from, and sustain participation in other public services and programmes.

Even well-designed nutrition and health programmes assume a baseline level of financial stability that many households do not possess. Without sufficient and predictable income, and competing financial demands such as housing costs, debt repayment, or irregular employment, individuals may be unable to act on nutrition advice, attend appointments, or maintain consistent participation in support schemes and community food initiatives. As such, by reducing financial precarity, UBI may improve the real-world effectiveness of existing interventions without altering their core design.

This systems logic aligns with evidence that food insecurity in the UK is shaped by complex, interacting drivers spanning welfare policy, labour markets, housing, and food systems, with responsibility dispersed across multiple government departments (29). Introducing a universal income floor could represent a partial reassertion of state responsibility for income adequacy, reducing reliance on charitable provision while enabling more coherent alignment between social security, public health, and food policy objectives.

Several policy combinations illustrate how UBI could function synergistically rather than competitively (see Table 1). Conceptualised through a systems lens, UBI does not represent a single-policy solution to food insecurity. Instead, it functions as an enabling condition, providing financial stability that allows individuals to navigate, access, and benefit from the wider policy environment. While it cannot replace targeted nutrition, labour, or food system specific interventions, it may make them more equitable, more effective, and more sustainable over time.

**Table 1:** Summary of how UBI could function synergistically rather than competitively with other policy options.

UBI + Additional policy	Possible outcome
UBI + free school meals	Address nutritional needs across both household and child-specific domains.
UBI + strong minimum wage	Dual mechanism for tackling in-work poverty reinforcing both welfare-state and labour-market institution: income security across employment statuses + labour-market fairness
UBI + nutrition-specific programmes	Address specific nutritional needs, particularly for infants, children, and pregnant women + reduce financial constraints, allowing targeted interventions to operate as intended rather than as compensatory mechanisms for wider social insecurity.
UBI + local food system investment	Increase households’ capacity to afford healthier, locally produced food + support more resilient and sustainable food economies, particularly when combined with public investment. Lower reliance of food growers on high-interest loans and government subsidies allowing for increased local food production

## 5 UBI as a long-term investment: economic, social, and health impacts

Whilst fiscal costs of UBI are a concern in policy debates, these discussions give limited attention to the economic costs associated with food insecurity and poverty. In the UK, diet-related ill health and malnutrition contribute to avoidable pressures on the National Health Service (NHS), while food insecurity is associated with poorer educational outcomes, reduced labour-force participation, and constrained long-term economic productivity (40, 41). UK-specific modelling studies suggest that income insecurity represents a significant and persistent public expenditure challenge (42, 43).

Widerquist and Arndt’s (43) microsimulation assessed both the gross and net costs of an anti-poverty level UBI, specifying both the payment levels and the income tax rate applied to beneficiaries. The authors determined not only the distributional impact of the scheme but also the financial outlay required for its delivery (44). Using data from the UK government’s 2019–2020 fiscal year, their analysis placed the gross cost of a UBI scheme at £486 billion, equivalent to 20.9% of GDP. However, after deductions are factored in (e.g., taxes for those who are financially wealthy), the true net cost of a UBI would be closer to £45 billion, a figure substantially lower than the corresponding gross cost projections. The authors also anticipated longer-term savings across all social policy domains as people are lifted out of poverty. Further micro-simulation and economic modelling by Reed et al. (42) goes further. Using data from the Family Resources Survey and the Landman Economics Tax-Transfer Model (TTM) the authors microsimulate the impacts of three UBI schemes (low, medium and high payments) and compare against the present UK tax-benefit system baseline. Reed and authors demonstrate that their ‘scheme 1’—a modest UBI scheme of £2,132 for children, £3,276 for adults and £9,880 for the elderly per year—could be implemented immediately, reduce child poverty to the lowest level achieved since 1961, and would be fiscally neutral, i.e., the cost of the scheme would be offset by extra revenue from changes in tax rates and National Insurance Contributions.

UBI may also generate wider economic and social benefits through its effects on local economies and community resilience, for example by increasing consumer spending on essential goods and services, supporting businesses and creating jobs (45). With greater consumer purchasing power, UBI could strengthen local economic activity and contribute to more resilient local food systems and labour markets. However, the extent to which these effects would materialise in the UK would depend on the amount of UBI given, interaction with existing welfare systems, and broader economic conditions.

Health-related pathways provide an additional lens through which to consider UBI as a potential long-term investment. By reducing financial stress, income security may improve mental wellbeing, which is closely linked to dietary behaviours, chronic disease risk, and healthcare utilisation. Improvements in diet quality and reduced reliance on coping strategies such as meal skipping or low-cost, energy-dense foods could plausibly contribute to better health trajectories over the life course (46). While such outcomes cannot be assumed, international evidence from unconditional cash transfer programmes suggests potential for improvements in wellbeing, nutrition, and health-related behaviours, particularly when income support is sustained over time.

## 6 Future directions for research and policy learning

If income security is to be taken seriously as a lever for improving food security and population health, future research and policy must move beyond abstract debates about feasibility and focus on evidence generation and policy learning.

For UK funders and policymakers, including the National Institute for Health and Care Research (NIHR), the Department of Health and Social Care (DHSC), and devolved administrations, this requires treating food and nutrition outcomes as core, rather than peripheral, considerations in income-based policy design.

Secondly, future UBI and UBI-adjacent pilots should explicitly and systematically measure food-related outcomes. Despite growing interest in guaranteed income schemes, and the importance of improving the affordability of food to communities (47), to the authors knowledge, no pilots (or their evaluations) have collected robust data on food insecurity, dietary quality, or food purchasing behaviours. In the recent evaluation report of the Welsh care leavers UBI pilot, food is mentioned throughout—but even here, it is done so in relation to mental or physical health rather than as a standalone metric or outcome (48). Embedding food insecurity, diet quality and food purchasing behaviours as outcomes in monitoring and evaluation of UBI schemes would allow policymakers to better understand the pathways through which income security influences diet and health, and to compare income-based approaches with food-specific interventions.

Thirdly, equity impacts must be central to future research agendas. Income-based policies are often presented as universally beneficial, yet their effects are likely to vary across population groups. Research should examine how UBI interacts with disability, caring responsibilities, ethnicity, migration status, and insecure legal or labour-market positions. Understanding which groups benefit most (for example, by working closely with communities), and which may require complementary support, is essential for designing inclusive welfare reforms that reduce, rather than reproduce, existing inequalities in food insecurity.

Lastly, greater attention should be given to combined and hybrid policy models. Rather than evaluating UBI in isolation, policymakers should assess how income guarantees interact with minimum wage policies, housing costs, childcare provision, and targeted nutrition programmes and recommend associated policies such as strengthening universal basic services, introducing rent caps and reduced childcare costs (44). Comparative research is needed to examine basic income guarantees, partial or negative income tax models, and mixed approaches that combine income floors with nutrition-specific services. Such work would help identify policy configurations that are both fiscally and socially effective in the UK context.

With reference to the prior suggestions, innovative methodology such as systems-thinking and modelling approaches is required. Food insecurity emerges from complex interactions between income, prices, food environments, social norms, and health (29). Employing participatory systems mapping techniques and quantitative simulation-based systems models could help researchers, policymakers and wider communities understand how unconditional income interventions interact with other food and nutrition-targeted policies, local food systems and supply chains over time, anticipate unintended consequences, and identify leverage points for more effective policy delivery under varying contextual settings within the UK (49). For NIHR and other research funders, investing in such approaches would strengthen the evidence base for integrated, upstream policy solutions.

## 7 Conclusion: reimagining food security through income security

The UK’s escalating food insecurity crisis reflects deep structural failures: insecure and inadequate incomes, rising living costs, welfare retrenchment, and fragmented policy responses. While emergency food aid and targeted nutrition programmes provide essential relief, they primarily treat the symptoms of food insecurity rather than its underlying cause. As welfare tightening continues to erode household budgets, the limitations of reactive, conditional approaches have become increasingly apparent.

UBI is not a panacea. Income support alone cannot address power imbalances within food systems, reshape unhealthy food environments, or resolve structural inequities in housing, labour markets, and food supply chains. However, the evidence reviewed in this article suggests that income security is a necessary precondition for food security. By providing a stable and unconditional income floor, UBI may reduce financial precarity, support healthier food choices, and enhance dignity and autonomy, while making existing nutrition and social policies more effective.

## Data Availability

The original contributions presented in the study are included in the article/supplementary material, further inquiries can be directed to the corresponding author.
